# Extinction and persistence of a stochastic SIRV epidemic model with nonlinear incidence rate

**DOI:** 10.1186/s13662-021-03347-3

**Published:** 2021-04-08

**Authors:** Ramziya Rifhat, Zhidong Teng, Chunxia Wang

**Affiliations:** 1 College of Medical Engineering and Technology, Xinjiang Medical University, Urumqi, 830017 P.R. China; 2 College of Mathematics and Systems Science, Xinjiang University, Urumqi, 830046 P.R. China

**Keywords:** 92D30, 60H10, 60H40, Stochastic epidemic model, Threshold value, Extinction, Permanence in the mean

## Abstract

In this paper, a stochastic SIRV epidemic model with general nonlinear incidence and vaccination is investigated. The value of our study lies in two aspects. Mathematically, with the help of Lyapunov function method and stochastic analysis theory, we obtain a stochastic threshold of the model that completely determines the extinction and persistence of the epidemic. Epidemiologically, we find that random fluctuations can suppress disease outbreak, which can provide us some useful control strategies to regulate disease dynamics. In other words, neglecting random perturbations overestimates the ability of the disease to spread. The numerical simulations are given to illustrate the main theoretical results.

## Introduction

Recent global infectious diseases (such as the outbreak of H7N9 influenza in 2013, Ebola disease in 2014, and COVID-19 in 2019) resulted in a lot of biological deaths and substantial financial ruins. Infectious diseases are a major concern of the public. The modeling of infection diseases is extremely important to research the mechanisms of diseases. A mathematical model is considered as an effective way to forecast the outbreak of a disease [1–17].

In fact, our real life is full of randomness and stochasticity. For human disease related epidemics, the nature of epidemic growth and spread is random due to the unpredictability in person to person contacts. Because of environmental noises, the deterministic approach has some limitations in the mathematical modeling transmission of an infectious disease, and several authors began to consider the effect of white noise on the epidemic systems. In order to improve the understanding of the difference of random environmental fluctuations, many scholars have introduced white noise in deterministic models [18–39].

There are different approaches used in the literature to introduce random perturbations into population models, both from a mathematical and biological perspective. One is to perturb the positive endemic equilibria in order to make the equilibria of deterministic models robust. In this situation, the essence of the investigation using the approach is to check if the asymptotic stability of the positive equilibria of deterministic models can be preserved. The other important approach is with parameter perturbation. Many literature works on this approach can be found, for example, [25–29]. In epidemic models, the natural death rate *d* and the disease transmission parameter *β* are two of the key parameters to disease transmission. May in [37] pointed out that all the parameters involved in the population model exhibit random fluctuation as the factors controlling them are not constant. And in the real situation, the natural death rate *d* and the disease transmission parameter *β* always fluctuate around some average value due to continuous fluctuation in the environment. In this sense, *d* can seem as a random variable *d̃*, *β* changes to a random variable *β̃*. More precisely, each infected individual makes $-\tilde{d}\,dt = -d \,dt + \sigma \,dB(t)$, $\tilde{\beta }\,dt = \beta \,dt + \sigma \,dB(t)$ potentially infectious contacts with each other individual in $[t,t + dt)$.

In recent years, the stochastic SIV and SIRV type epidemic models have been extensively studied, and many important results have been established, see, for example, articles [18–24, 40–43] and the references cited therein. We easily see that most of these research works aim at the models with bilinear incidence, and there exists some research on the models with special nonlinear incidences (see [19–21]). Particularly in [20], the authors studied a class of stochastic SIVS epidemic models with nonlinear saturated incidence:

$$ \textstyle\begin{cases} dS= [ (1-q)\Lambda -(\mu +p) S- \frac{\beta SI}{\psi (I)}+\gamma I+\delta V]\,dt - \frac{\sigma SI}{\psi (I)}\,dB, \\ dI= [\frac{\beta SI}{\psi (I)}-(\mu + \gamma +\nu )I]\,dt+\frac{\sigma SI}{\psi (I)}\,dB, \\ dV= [q \Lambda +pS-(\mu +\delta )V]\,dt. \end{cases} $$

A threshold value $\tilde{R}_{0}$ is identified. It is shown that if $\tilde{R}_{0}<1$, then the disease in the stochastic model is extinct, and if $\tilde{R}_{0}>1$, then any solution with positive initial value in $R_{+}^{3}$ is permanent in the mean. In [21], the authors studied a class of stochastic SIVR epidemic models where vaccination is included and such that the immunity is permanent, respectively:

$$ \textstyle\begin{cases} dS= [\mu -\beta SI-(\mu +\phi )S]\,dt-\sigma SI\,dB, \\ dI= [\beta SI+\rho \beta VI-(\lambda +\mu )I]\,dt+\sigma (S+ \rho V)I \,dB, \\ dV= [\phi S-\rho \beta V I-\mu V]\,dt-\rho \sigma V I\,dB, \\ dR=(\lambda I-\mu R)\,dt. \end{cases} $$

The sufficient conditions on the exponential stability in mean square of disease-free equilibrium are obtained.

Motivated by the above work, in this paper, we consider the following deterministic SIRV epidemic model with nonlinear incidence rate and disease-induced mortality:

1.1 $$ \textstyle\begin{cases} dS= [ (1-\epsilon )\Lambda -(\mu +q) S- \beta f(S,I)+\eta V]\,dt, \\ dI= [\beta f(S,I)-(\mu +\gamma +\alpha )I]\,dt, \\ dR= [\gamma I -\mu R]\,dt, \\ dV= [\epsilon \Lambda +qS-(\mu +\eta )V]\,dt. \end{cases} $$

In model (1.1), the basic reproduction number $R_{0}= \frac{\beta \frac{\partial f(S^{0},0)}{\partial I}}{\mu +\gamma +\alpha }$ is a threshold which completely determines the persistence or extinction of the disease. It is shown that, if $R_{0}\leq 1$, then the disease-free equilibrium $E _{0}$ is globally asymptotically stable, and if $R_{0}>1$, then model (1.1) has a unique endemic equilibrium $E^{ *}$ which is globally asymptotically stable.

Now, we assume that the random effects of the environment make the transmission coefficient *β* of the disease in deterministic model (1.1) generate random disturbance. That is, $\beta \to \beta +\sigma \dot{B}(t)$, where $B(t)$ is a one-dimensional standard Brownian motion defined on some probability space. Thus, model (1.1) will become the following stochastic SIRV epidemic model with nonlinear incidence rate:

1.2 $$ \textstyle\begin{cases} dS= [ (1-\epsilon )\Lambda -(\mu +q) S- \beta f(S,I)+\eta V]\,dt -\sigma f(S,I)\,dB, \\ dI= [\beta f(S,I)-(\mu +\gamma +\alpha )I]\,dt+ \sigma f(S,I)\,dB, \\ dR= [\gamma I -\mu R]\,dt, \\ dV= [\epsilon \Lambda +qS-(\mu +\eta )V]\,dt. \end{cases} $$

The biological meaning of all parameters in (1.2) is the same as that in system (1) in [18]. All parameter values are assumed to be nonnegative and *λ*, $\mu >0$. The portion *ϵ*Λ ($0 \leq \epsilon \leq 1$) is vaccinated, whereas the rest $(1-\epsilon )\Lambda $ remains in the susceptible class.

As well as we know, in modeling the dynamics of epidemic systems the incidence rate is an important substance. In practice the nonlinear incidence is frequently used for achieving more exact results. We see that some deterministic and stochastic epidemic models with nonlinear incidence have been extensively studied (see, for example, [30–35, 38–44]). However, we see that stochastic epidemic models with nonlinear incidence and vaccination are barely studied. Therefore, our first question is: Can we also establish a series of similar results on the extinction (i.e., disease-free) or persistence (i.e., endemic) of the disease for the stochastic SIRV epidemic model with nonlinear incidence and vaccination?

The main focus of this article is to investigate how environment fluctuations affect disease dynamics through studying the global dynamics of an SIRV model with nonlinear incidence in both the deterministic and the corresponding stochastic version. The organization of this paper is as follows. In Sect. 2, we give some useful lemmas and fundamental assumption for general nonlinear incidence functions. In Sect. 3, the results on the extinction of the disease with probability one are stated and proved. In Sect. 4, we prove that the disease is persistent under one condition. In Sect. 5, the numerical simulations are presented. Finally, in Sect. 6, a conclusion is given.

## Preliminaries

Denote $R_{+}^{4}=\{(x_{1},x_{2},x_{3},x_{4}): x_{i}\geq 0,i=1,2,3,4\}$. For any integrable function $g(t)$ defined for $t\geq 0$, we denote $\langle g\rangle _{t}=\frac{1}{t}\int _{0}^{t}g(s)\,ds$. The initial condition for model (1.2) is given by

2.1 $$ S(0)=S_{0},\qquad I(0)=I_{0} ,\qquad R(0)=R_{0},\qquad V(0)=V_{0}, $$

where $(S_{0},I_{0},R_{0},V_{0})\in R_{+}^{4}$. Moreover, for a nonlinear function $f(S,I)$ in model (1.2), we always introduce the following assumptions (see [34]).

*(H)* The function $f (S,I)$ is two-order continuously differentiable for any $S,I\geq 0$ with $S+I>0$, strictly monotone increasing for $S\geq 0$ for any fixed $I>0$, and monotone increasing for $I > 0$ for any fixed $S\geq 0$. Moreover, the function $\tilde{\xi }(S,I)\triangleq \frac{f(S,I)}{I}$ is bounded and monotone decreasing for $I > 0$ for any fixed $S\geq 0$, and $f(0,I) = f (S, 0) = 0$ for all $S,I >0$.

### Remark 2.1

Define $\tilde{\zeta }(S)\triangleq \frac{\partial f(S,0)}{\partial I}$, from assumption $(H)$, $\tilde{\zeta }(S)$ is continuous and monotone increasing for $S\geq 0$. By simple calculation, we can obtain $0 \leq \tilde{\xi }(S,I)\leq \tilde{\zeta }(S) $ for any $S > 0$ and $I > 0$.

### Lemma 2.2

*For any initial value* $(S_{0},I_{0},R_{0},V_{0})\in R_{+}^{4}$, *model* (1.2) *has a unique solution* $(S(t),I(t),R(t),V(t))$ *with initial condition* (2.1) *defined for all* $t\geq 0$, *and the solution remains in* $R_{+}^{4}$ *with probability one for any* $t\geq 0$. *Furthermore*,

$$ \frac{\Lambda }{\mu +\alpha }\leq \liminf_{t\to \infty }N(t)\leq \limsup _{t\to \infty }N(t)\leq \frac{\Lambda }{\mu } \quad \textit{a.s.}, $$

*where* $N(t)=S(t)+I(t)+R(t)+V(t)$.

### Proof

Since the coefficients of model (1.2) are locally Lipschitz continuous, by the fundamental theory of stochastic differential equations, for any initial value $(S_{0},I_{0},R_{0},V_{0})\in \mathbb{R}_{+}^{4}$, model (1.2) has a unique local solution $(S(t),I(t),R(t),V(t))$ defined for $t\in [0,\tau _{e})$ and satisfies $(S(t),I(t),R(t),V(t))\in \mathbb{R}_{+}^{4}$ a.s. for all $t\in [0,\tau _{e})$, where $\tau _{e}$ is the explosion time (see [36]). Let $N(t)=S(t)+I(t)+R(t)+V(t)$, then we have

2.2 $$ d N(t)=\bigl[\Lambda -\mu N(t)-\alpha I(t)\bigr]\,dt. $$

Consequently,

2.3 $$ \bigl[\Lambda -(\mu +\alpha )N(t)\bigr]\,dt\leq d N(t)\leq \bigl[ \Lambda -\mu N(t)\bigr]\,dt. $$

Therefore, we further have, for any $t\in [0,\tau _{e})$,

2.4 $$ \max \biggl\{ N(0),\frac{\Lambda }{\mu +\alpha }\biggr\} \leq N(t)\leq \max \biggl\{ N(0), \frac{\Lambda }{\mu }\biggr\} \triangleq \bar{M} \quad \mbox{a.s..} $$

This shows that $0\leq S(t)$, $I(t), V(t), R(t)\leq \bar{M}$ a.s. for all $t\in [0,\tau _{e})$. It follows from assumption $(H)$ that there is a constant $M_{0}>0$ such that $\max_{t\in [0,\tau _{e})}\{\frac{f(S(t),I(t))}{S(t)}\}\leq M_{0}$ a.s..

To show that the solution is global, we only need to prove that $\tau _{e}=\infty $ a.s. Let $k_{0}\geq 0$ be large enough such that $(S_{0},I_{0},R_{0},V_{0})$ all lie within the interval $[\frac{1}{k_{0}},\bar{M}]$. For each integer $k\geq {k_{0}}$, define the following stopping time:

$$ \begin{aligned} \tau _{k}&=\inf \biggl\{ t\in [0,\tau _{e}): \min \bigl\{ S(t),I(t),R(t),V(t)\bigr\} \leq \frac{1}{k} \biggr\} . \end{aligned} $$

Throughout this paper, we set $\inf {\O }{=}\infty $, where Ø denotes the empty set. It is clear that $\tau _{k}$ is increasing as $k\rightarrow \infty $. Set $\tau _{\infty }=\lim_{k\rightarrow \infty }\tau _{k}$, then $\tau _{\infty }\leq \tau _{e} $ a.s. Namely, we need to show that $\tau _{\infty }{=}\infty $ a.s. Assume that there exist a pair of constants $T>0$ and $\epsilon \in (0,1)$ such that $P\{\tau _{\infty }\leq T\}>\epsilon $. Then there is an integer $k_{1}\geq {k_{0}}$ such that, for all $k\geq {k_{1}}$,

2.5 $$ \begin{aligned} P\{\tau _{k}\leq T\}>\epsilon . \end{aligned} $$

Define a $C^{2}$-function as follows:

$$ V(t)=(S-1-\log S)+ (I-1-\log I)+ (R-1-\log R)+ (V-1-\log V). $$

The nonnegativity of $V(t)$ can be seen from $u-1-\log u\geq 0$ for $u\geq 0$. Using Itô’s formula (see [45]), we obtain

$$ \begin{aligned} dV(t)=LV(t)\,dt+\sigma \biggl[ \frac{f(S,I)}{S}- \frac{f(S,I)}{I}\biggr]\,dB(t), \end{aligned} $$

where

$$ \begin{aligned} LV(t)&=\biggl(1-\frac{1}{S}\biggr) \bigl((1- \epsilon )\Lambda -(\mu +q) S-\beta f(S,I)+ \eta V\bigr) \\ &\quad{}+\biggl(1-\frac{1}{I}\biggr) \bigl(\beta f(S,I)-(\mu +\gamma +\alpha )I \bigr)+ \frac{\sigma ^{2}f^{2}(S,I)}{2S^{2}}+ \frac{\sigma ^{2}f^{2}(S,I)}{2I^{2}} \\ &\quad{}+\biggl(1-\frac{1}{R}\biggr) (\gamma I -\mu R) +\biggl(1- \frac{1}{V}\biggr) \bigl(\epsilon \Lambda +qS-(\mu +\eta )V\bigr) \\ &= \Lambda -\mu (S+I+R+V)-\alpha I-\frac{(1-\epsilon )\Lambda }{S}+( \mu +q)+ \beta \frac{f(S,I)}{S} \\ &\quad{}-\eta \frac{V}{S}-\beta \frac{f(S,I)}{I}+(\mu +\gamma +\alpha )- \gamma \frac{I}{R}+\mu -\frac{\epsilon \Lambda }{V}-q\frac{S}{V}+(\mu + \eta ) \\ &\quad{}+\frac{\sigma ^{2}f^{2}(S,I)}{2S^{2}}+ \frac{\sigma ^{2}f^{2}(S,I)}{2I^{2}} \\ &\leq \Lambda +4\mu +q+\gamma +\alpha +\eta +\beta \frac{f(S,I)}{S}+ \frac{\sigma ^{2}f^{2}(S,I)}{2S^{2}}+ \frac{\sigma ^{2}f^{2}(S,I)}{2I^{2}}. \end{aligned} $$

Clearly, we further have

$$ \begin{aligned} LV(t)&\leq \Lambda +4\mu +q+\gamma +\alpha +\eta + \beta M_{0}+ \frac{\sigma ^{2} M_{0}^{2} }{2}+\frac{\sigma ^{2}}{2}\biggl( \frac{ \partial f(S^{0},0)}{\partial I}\biggr)^{2}\triangleq B, \end{aligned} $$

where $S^{0}=\frac{\Lambda [\mu (1-\epsilon )+\eta ]}{\mu (\mu +\eta +q)}$. Therefore, we have

2.6 $$ \begin{aligned} dV(t)\leq B\,dt+\sigma \biggl[ \frac{f(S,I)}{S}- \frac{f(S,I)}{I}\biggr]\,dB(t). \end{aligned} $$

Integrating (2.6) from 0 to $T\wedge {\tau _{k}}$ and then taking expectations, we can obtain

2.7 $$ \begin{aligned} \mathbb{E}V\bigl(S(T\wedge {\tau _{k}}),I(T \wedge {\tau _{k}}),R(T\wedge { \tau _{k}}),V(T\wedge {\tau _{k}})\bigr) \leq {V(S_{0},I_{0},R_{0},V_{0})}+BT< \infty . \end{aligned} $$

Set $\Omega _{k}=\{\tau _{k}\leq T\}$ for $k\geq {k_{1}}$, then $P\{\Omega _{k}\}\geq \epsilon $ by (2.5). Noticing that, for every $\omega \in \Omega _{k}$, there is at least one of $S(\tau _{k},\omega )$, $I(\tau _{k},\omega )$, $R(\tau _{k},\omega )$, $V(\tau _{k},\omega )$ that equals to $\frac{1}{k}$. Hence,

2.8 $$ \begin{aligned} V\bigl(S(\tau _{k},\omega ),I(\tau _{k},\omega ),R(\tau _{k},\omega ),V( \tau _{k}, \omega )\bigr)&\geq \frac{1}{k}-1+\log {k}. \end{aligned} $$

In view of (2.7) and (2.8), we have

$$ \begin{aligned} V(S_{0},I_{0},R_{0},V_{0})+BT &\geq {\mathbb{E}}\bigl[I_{\Omega _{k}}V\bigl(S( \tau _{k},\omega ),I(\tau _{k},\omega ),R(\tau _{k},\omega ),V(\tau _{k}, \omega )\bigr)\bigr] \\ &\geq \varepsilon \biggl(\frac{1}{k}-1+\log {k}\biggr), \end{aligned} $$

where $I_{\Omega _{k}}$ is the indicator function of $\Omega _{k}$. Let $k\rightarrow \infty $ lead to the contradiction

$$ \infty >V(S_{0},I_{0},R_{0},V_{0})+BT= \infty . $$

Therefore, we must have $\tau _{\infty }=\infty $ a.s..

Furthermore, since (2.3) and (2.4) hold for all $t\in [0,\infty )$, we can obtain

$$ \frac{\Lambda }{\mu +\alpha }\leq \liminf_{t\to \infty }N(t)\leq \limsup _{t\to \infty }N(t)\leq \frac{\Lambda }{\mu } \quad \mbox{a.s.}, $$

and when $N(0)\in [\frac{\lambda }{\mu +\alpha },\frac{\Lambda }{\mu }]$ we also have $N(t)\in [\frac{\lambda }{\mu +\alpha },\frac{\Lambda }{\mu }]$ a.s. for all $t\in [0,\infty )$. This competes the proof. □

### Remark 2.3

Denote the region

$$ \Gamma =\biggl\{ (S,I,R,V): S\geq 0,I\geq 0,R\geq 0,V\geq 0, \frac{\Lambda }{\mu +\alpha } \leq S+I+R+V\leq \frac{\Lambda }{\mu }\biggr\} . $$

The proof of Lemma 2.2 shows that Γ is globally attractive and positive invariant with respect to model (1.2) with probability one. Therefore, in the following discussions we can assume that the initial value $(S_{0},I_{0},R_{0},V_{0})\in \Gamma $ for any solution $(S(t),I(t),R(t),V(t))$ of model (1.2).

### Lemma 2.4

*Let* $(S(t),I(t),R(t),V(t))$ *be the solution of model* (1.2) *with initial value* $(S(0),I(0),R(0),V(0))\in \Gamma $. *Then*

2.9 $$ S(t)=S^{0}+H_{1}(t)+G(t), $$

*where*

$$ H_{1}(t)=\tilde{\eta }e^{-(\mu +\eta +q)t} +\tilde{H}_{0}e^{-\mu t} \frac{\eta }{q +\eta }-\biggl(V(0)-\frac{\epsilon \Lambda }{\mu +\eta }\biggr)e^{-(\mu +\eta +q)t} - \tilde{H}_{0}e^{-(\mu +\eta +q)t}\frac{q}{\eta +q}, $$

$\tilde{H}_{0}=\frac{\Lambda }{\mu }-N(0)+R(0)$, $\tilde{\eta }= \frac{q\Lambda [(1-\epsilon )\mu +\eta ]}{\mu (\mu +\eta )(\gamma +\eta +q)}$, *and*

$$ \begin{aligned} G(t)&= -I(t)-(\alpha +\gamma ) \int _{0}^{t}e^{-\mu (t-s)}I(s)\,ds+q \int _{0}^{t}e^{-(\mu +\eta +q)(t-s)}I(s)\,ds \\ &\quad{}+ q(\alpha +\gamma ) \int _{0}^{t}e^{-(\mu +\eta +q)(t-s)} \int _{0}^{s}e^{- \mu (s-u)}I(u)\,du \,ds. \end{aligned} $$

*Furthermore*,

2.10 $$ \langle S\rangle _{t}= S^{0}- \frac{(\mu +\eta )(\mu +\alpha +\gamma )}{\mu (\mu +\eta +q)}\langle I \rangle _{t}-\varphi (t), $$

*where*

2.11 $$ \varphi (t)= \frac{(\mu +\eta )[N(t)-N(0)]-\mu (V(t)-V(0))-(\mu +\eta )(R(t)-R(0))}{\mu (\mu +\eta +q)t}. $$

### Proof

From (2.2) and model (1.2), we can obtain

2.12 $$\begin{aligned}& N(t)=\frac{\Lambda }{\mu }+\biggl[N(0)-\frac{\Lambda }{\mu } \biggr]e^{-\mu t}-\alpha \int ^{t}_{0}e^{-\mu (t-s)}I(s)\,ds, \end{aligned}$$

2.13 $$\begin{aligned}& V(t)=\frac{\epsilon \Lambda }{\mu +\eta }+\biggl(V(0)- \frac{\epsilon \Lambda }{\mu +\eta } \biggr)e^{-(\mu +\eta )t}+q \int ^{t}_{0}S(s)e^{-( \mu +\eta )(t-s)}\,ds, \end{aligned}$$

and

2.14 $$ R(t)=R(0)e^{-\mu t}+\gamma \int ^{t}_{0}I(s)e^{-\mu (t-s)}\,ds. $$

Combining with (2.12), (2.13), and (2.14), we can obtain

2.15 $$ S(t)=\tilde{A}-H(t)-I(t)-(\alpha +\gamma ) \int ^{t}_{0}e^{-\mu (t-s)}I(s)\,ds - q \int ^{t}_{0}S(s)e^{-(\mu +\eta )(t-s)}\,ds, $$

where

$$ H(t)=\biggl(V(0)-\frac{\epsilon \Lambda }{\mu +\eta }\biggr)e^{-(\mu +\eta )t}+R(0)e^{- \mu t}+ \biggl(\frac{\Lambda }{\mu }-N(0)\biggr)e^{-\mu t} $$

and $\tilde{A}=\frac{\Lambda [(1-\epsilon )\mu +\eta ]}{\mu (\mu +\eta )}$.

By (2.15), we further have

2.16 $$ \begin{aligned} &d\biggl[e^{q t} \int ^{t}_{0}S(s)e^{(\mu +\eta )s}\,ds\biggr] \\ &\quad = \biggl\{ \tilde{A}e^{(\mu +\eta )t}e^{q t}-H(t)e^{(\mu + \eta )t}e^{q t}-I(t)e^{(\mu +\eta )t}e^{q t}- \alpha e^{(\mu +\eta )t}e^{q t} \int ^{t}_{0}e^{-\mu (t-s)}I(s)\,ds \\ &\qquad{}- \gamma e^{(\mu +\eta )t}e^{q t} \int ^{t}_{0}I(s)e^{- \mu (t-s)} \,ds+e^{q t}q \int ^{t}_{0}S(s)e^{(\mu +\eta )s}\,ds \\ &\qquad{}- e^{(\mu +\eta )t}e^{q t}p \int ^{t}_{0}S(s)e^{-(\mu +\eta )(t-s)}\,ds \biggr\} \,dt. \end{aligned} $$

Integrating (2.16) from 0 to *t* and multiplying by $e^{-(\mu +\eta )t}$, we can obtain

2.17 $$ \begin{aligned} & \int ^{t}_{0}S(s)e^{-(\mu +\eta )(t-s)}\,ds \\ &\quad = S^{0}\bigl(1-e^{-(\mu +\eta +q)t}\bigr) - \int ^{t}_{0}H(s)e^{-( \mu +\eta +q)(t-s)}\,ds \\ &\qquad{}- \int ^{t}_{0}I(s)e^{-(\mu +\eta +q)(t-s)}\,ds -\alpha \int ^{t}_{0}e^{-(\mu +\eta +q)(t-s)} \int ^{s}_{0}e^{-\mu (s-u)}I(u)\,du \,ds \\ &\qquad{}- \gamma \int ^{t}_{0}e^{-(\mu +\eta +q)(t-s)} \int ^{s}_{0}e^{- \mu (s-u)}I(u)\,du \,ds. \end{aligned} $$

By substituting (2.17) into (2.15), we further obtain (2.9).

Next, we prove (2.10), from (2.2) and model (1.2) we further have

$$\begin{aligned}& \frac{1}{t}\bigl(N(t)-N(0)\bigr)=\Lambda -\mu \langle S\rangle _{t}-(\mu + \alpha )\langle I\rangle _{t}-\mu \langle R \rangle _{t}-\mu \langle V \rangle _{t}, \\& \frac{1}{t}\bigl(R(t)-R(0)\bigr)=\gamma \langle I\rangle _{t}- \mu \langle R \rangle _{t}, \end{aligned}$$

and

$$ \frac{1}{t}\bigl(V(t)-V(0)\bigr)=\epsilon \Lambda +q\langle S\rangle _{t}-(\mu + \eta )\langle V\rangle _{t}. $$

Consequently,

$$ \begin{aligned} & \frac{1}{t}\bigl[(\mu +\eta ) (N(t)-N(0) \bigr]-(\mu +\eta ) \bigl(R(t)-R(0)\bigr)- \mu \bigl(V(t)-V(0)\bigr) \\ &\quad = \bigl[(\mu +\eta )\Lambda -\mu \epsilon \Lambda -\mu (\mu + \eta )\langle S \rangle _{t}-\mu q\langle S\rangle _{t}-(\mu +\alpha ) ( \mu + \eta )\langle I\rangle _{t}-(\mu +\eta )\gamma \langle I\rangle _{t}\bigr]. \end{aligned} $$

Therefore,

$$ \begin{aligned} &\bigl(\mu (\mu +\eta )+\mu q\bigr)\langle S\rangle _{t} \\ &\quad =(\mu +\eta )\Lambda -\mu \epsilon \Lambda -\bigl((\mu +\alpha ) (\mu + \eta )+(\mu +\eta )\gamma \bigr)\langle I\rangle _{t} \\ &\qquad{}-\frac{1}{t}\bigl[(\mu +\eta ) \bigl(N(t)-N(0)\bigr)-(\mu +\eta ) \bigl(R(t)-R(0)\bigr)-\mu \bigl(V(t)-V(0)\bigr)\bigr]. \end{aligned} $$

Thus, we finally obtain (2.10). This completes the proof. □

## Extinction of the disease

Define the parameter

$$ \widetilde{R}_{0}= \frac{\beta \tilde{\zeta }(S^{0})}{\mu +\gamma +\alpha }- \frac{\sigma ^{2}(\tilde{\zeta }(S^{0}))^{2}}{2(\mu +\gamma +\alpha )} = R_{0}- \frac{\sigma ^{2}(\tilde{\zeta }(S^{0}))^{2}}{2(\mu +\gamma +\alpha )}, $$

where we can easily see that $R_{0}$ is the basic reproduction number of deterministic model (1.1). On the extinction of the disease in probability for model (1.2) we have the following result.

### Theorem 3.1

*Let* $(S(t),I(t),R(t),V(t))$ *be any solution of model* (1.2) *with initial value* $(S(0),I(0),R(0),V(0)) \in \Gamma $. *Assume that one of the following conditions holds*:

$(a)$ $\widetilde{R}_{0}<1$ *and* $\frac{\partial ^{2} f(S,0)}{\partial S \partial I}$ *is decreasing for* $S>0$; $(b)$ $\sigma ^{2}>\frac{\beta ^{2}}{2(\mu +\gamma +\alpha )}$.

*Then we have*

$$ \begin{aligned} &\limsup_{t\rightarrow \infty }\frac{\log I(t)}{t} \leq (\mu +\gamma + \alpha ) (\tilde{R}_{0}-1)< 0 \quad \textit{a.s. if condition (a) holds}; \\ &\limsup_{t\rightarrow \infty }\frac{\log I(t)}{t}\leq \frac{\beta ^{2}}{2\sigma ^{2}}-(\mu + \gamma +\alpha )< 0 \quad \textit{a.s. if condition (b) holds}. \end{aligned} $$

### Proof

Using Itô’s formula to $\ln I(t)$, and then integrating from 0 to *t* for any $t>0$, we can obtain

3.1 $$ \begin{aligned} \frac{1}{t}\log \frac{I(t)}{I(0)}&= \beta \bigl\langle \tilde{\xi }(S,I)\bigr\rangle _{t}-(\mu +\gamma +\alpha )- \frac{\sigma ^{2}}{2} \bigl\langle \bigl( \tilde{\xi }(S,I)\bigr)^{2}\bigr\rangle _{t}+ \frac{\sigma }{t} \int _{0}^{t}\tilde{\xi }\bigl(S(s),I(s)\bigr) \,dB(s). \end{aligned} $$

By the Cauchy–Schwarz inequality, we further have

3.2 $$ \begin{aligned} \frac{1}{t}\log \frac{I(t)}{I(0)}&\leq \beta \bigl\langle \tilde{\xi }(S,I)\bigr\rangle _{t}-(\mu +\gamma +\alpha )- \frac{\sigma ^{2}}{2} \bigl\langle \tilde{\xi }(S,I)\bigr\rangle _{t}^{2}+ \frac{\sigma }{t} \int _{0}^{t}\tilde{\xi }\bigl(S(s),I(s)\bigr) \,dB(s). \end{aligned} $$

If condition $(a)$ holds, we further have

3.3 $$ \begin{aligned} \frac{1}{t}\log \frac{I(t)}{I(0)}&\leq \beta \bigl[\bigl\langle \tilde{\xi }(S,I)\bigr\rangle _{t}+\varphi (t)\bigr]-(\mu + \gamma +\alpha )-\frac{\sigma ^{2}}{2} \bigl[\bigl\langle \tilde{\xi }(S,I) \bigr\rangle _{t}+\varphi (t) \bigr]^{2} \\ &\quad{}-\Phi (t)+\frac{\sigma }{t} \int _{0}^{t}\tilde{\xi }\bigl(S(s),I(s)\bigr) \,dB(s), \end{aligned} $$

where

$$ \Phi (t)= \beta \varphi (t)-\frac{\sigma ^{2}}{2}\bigl[2 \bigl\langle \tilde{\xi }(S,I)\bigr\rangle _{t}\varphi (t)+\varphi ^{2}(t)\bigr]. $$

By the mean value theorem, we have

3.4 $$ \tilde{\xi }(S,I)=\tilde{\zeta }\bigl(S^{0}\bigr)+ \frac{\partial \tilde{\zeta }(\xi )}{\partial S}\bigl(S-S^{0}\bigr), $$

where *ξ* is situated between $S^{0}$ and *S*. Since $\frac{\partial \tilde{\zeta }(S)}{\partial S}\triangleq \frac{\partial ^{2} f(S,0)}{\partial S \partial I}$ is decreasing for $S>0$, if $S>S^{0}$, then $\xi \in (S^{0},S)$, we obtain

3.5 $$ \frac{\partial ^{2} f(\xi ,0)}{\partial S \partial I}\bigl(S-S^{0}\bigr)\leq \frac{\partial ^{2} f(S^{0},0)}{\partial S \partial I}\bigl(S-S^{0}\bigr), $$

and if $S\leq S^{0}$, then $\xi \in (S,S^{0})$, we also have

3.6 $$ \frac{\partial ^{2} f(\xi ,0)}{\partial S \partial I}\bigl(S-S^{0}\bigr)\leq \frac{\partial ^{2} f(S^{0},0)}{\partial S \partial I}\bigl(S-S^{0}\bigr). $$

Substituting (3.5) and (3.6) into (3.4) yields that

3.7 $$ \tilde{\xi }(S,I) \leq \tilde{\zeta }\bigl(S^{0}\bigr)+ \frac{\partial \tilde{\zeta }(S^{0})}{\partial S}\bigl(S-S^{0}\bigr). $$

Substituting (2.10) into (3.7), we further get

$$ \bigl\langle \tilde{\xi }(S,I)\bigr\rangle _{t}\leq \tilde{\zeta }\bigl(S^{0}\bigr)- \frac{\partial \tilde{\zeta }(S^{0})}{\partial S} \biggl( \frac{(\mu +\eta )(\mu +\alpha +\gamma )}{\mu (\mu +\eta +q)}\langle I \rangle _{t}+\varphi (t)\biggr). $$

Since $\frac{\partial \tilde{\zeta }(S^{0})}{\partial S}\triangleq \frac{\partial ^{2} f(S^{0},0)}{\partial S \partial I}\geq 0$ by assumption $(H)$, we hence have

3.8 $$ \bigl\langle \tilde{\xi }(S,I)\bigr\rangle _{t}+\varphi (t)\leq \tilde{\zeta }\bigl(S^{0}\bigr)+\biggl(1- \frac{\partial \tilde{\zeta }(S^{0})}{\partial S} \biggr)\varphi (t). $$

We obtain immediately from substituting (3.8) into (3.3) that

$$ \begin{aligned} \frac{1}{t}\log \frac{I(t)}{I(0)}&\leq \beta \tilde{\zeta }\bigl(S^{0}\bigr)-(\mu +\alpha +\gamma )- \frac{\sigma ^{2}}{2}\bigl( \tilde{\zeta }\bigl(S^{0}\bigr) \bigr)^{2}-\Phi (t) \\ &\quad{}+\frac{\sigma }{t} \int _{0}^{t}\tilde{\xi }\bigl(S(s),I(s)\bigr) \,dB(s)+ \Psi (t), \end{aligned} $$

where

$$ \begin{aligned} \Psi (t)&= \beta \biggl(1- \frac{\partial \tilde{\zeta }(S^{0})}{\partial S}\biggr) \varphi (t)-\sigma ^{2} \tilde{\zeta }\bigl(S^{0}\bigr) \biggl(1- \frac{\partial \tilde{\zeta }(S^{0})}{\partial S}\biggr)\varphi (t)- \frac{\sigma ^{2}}{2}\biggl(1- \frac{\partial \tilde{\zeta }(S^{0})}{\partial S}\varphi (t)\biggr)^{2}. \end{aligned} $$

From Lemma 2.2 and expression (2.11) of $\varphi (t)$, we can obtain $\lim_{t\rightarrow \infty }\varphi (t)=0$ a.s., which implies that $\lim_{t\rightarrow \infty }\Phi (t)=0$ and $\lim_{t\rightarrow \infty }\Psi (t)=0$ a.s.. Therefore, by the large number theorem for martingales, we finally have

3.9 $$ \limsup_{t\rightarrow \infty }\frac{\log I(t)}{t}\leq (\mu + \gamma + \alpha ) (\tilde{R}_{0}-1)< 0 \quad \mbox{a.s..} $$

If condition $(b)$ holds, then from (3.2) we have

$$ \frac{\log I(t)}{t}\leq \frac{\log I(0)}{t}+ \frac{\beta ^{2}}{2\sigma ^{2}}-(\mu +\gamma + \alpha ) + \frac{\sigma }{t} \int _{0}^{t}\tilde{\xi }\bigl(S(s),I(s)\bigr) \,dB(s). $$

With the large number theorem for martingales, we obtain

3.10 $$ \limsup_{t\rightarrow \infty }\frac{\log I(t)}{t}\leq \frac{\beta ^{2}}{2\sigma ^{2}}-(\mu +\gamma +\alpha )< 0 \quad \mbox{a.s..} $$

This completes the proof. □

### Theorem 3.2

*Assume that the conditions of Theorem *3.1*hold*. *Then*, *for any solution* $(S(t),I(t),R(t),V(t))$ *of model* (1.2) *with initial value* $(S(0),I(0),R(0),V(0)) \in \Gamma $, *we have*

$$ \lim_{t\to \infty }\bigl(S(t),I(t),R(t),V(t)\bigr)= \bigl(S^{0},0,0,V^{0}\bigr) \quad \textit{a.s.}, $$

*where* $V^{0}=\frac{\Lambda (\mu \epsilon +q)}{\mu (\mu +\eta +q)}$.

### Proof

From (3.9) and (3.10) we easily obtain $\lim_{t\rightarrow \infty }I(t)=0 $ a.s. Now, let us prove the assertion $S(t)\rightarrow S^{0}$ a.s., $V(t)\rightarrow V^{0}$ a.s., and $R(t)\rightarrow 0$ a.s. as $t\rightarrow +\infty $. According to (2.10) we get

$$ \lim_{t\rightarrow \infty }S(t)=S^{0}+\lim_{t\rightarrow \infty }H_{1}(t)+ \lim_{t\rightarrow \infty }G(t). $$

Clearly, $\lim_{t\rightarrow \infty }H_{1}(t)=0$, and

3.11 $$ \begin{aligned} \lim_{t\rightarrow \infty }G(t)&=\lim _{t \rightarrow \infty }\biggl\{ -I(t)-(\alpha +\gamma ) \int _{0}^{t}e^{-\mu (t-s)}I(s)\,ds+q \int _{0}^{t}e^{-(\mu +\eta +q)(t-s)}I(s)\,ds \\ &\quad{}+ q(\alpha +\gamma ) \int _{0}^{t}e^{-(\mu +\eta +q)(t-s)} \int _{0}^{s}e^{-\mu (s-u)}I(u)\,du \,ds\biggr\} . \end{aligned} $$

Using L’Hospital’s rule, we compute to obtain

3.12 $$\begin{aligned}& \begin{aligned} &\lim_{t\rightarrow \infty }(\alpha + \gamma ) \int _{0}^{t}e^{- \mu (t-s)}I(s)\,ds \\ &\quad = \lim_{t\rightarrow \infty }(\alpha +\gamma ) e^{- \mu t} \int _{0}^{t}e^{\mu s}I(s)\,ds =\alpha \lim _{t\rightarrow \infty } \frac{\int _{0}^{t}e^{\mu s}I(s)\,ds}{e^{\mu t}} \\ &\quad = (\alpha +\gamma )\lim_{t\rightarrow \infty } \frac{e^{\mu t}I(t)}{e^{\mu t}\mu }= \frac{(\alpha +\gamma )}{\mu }\lim_{t \rightarrow \infty }I(t)=0, \end{aligned} \end{aligned}$$

3.13 $$\begin{aligned}& \begin{aligned} & \lim_{t\rightarrow \infty }e^{-(\mu +\eta +q)t}q \int _{0}^{t}e^{(\mu +\eta +q)s}I(s)\,ds =p\lim _{t\rightarrow \infty } \frac{\int _{0}^{t}e^{(\mu +\eta +q)s}I(s)\,ds}{e^{(\mu +\eta +q)t}} \\ &\quad = q\lim_{t\rightarrow \infty } \frac{e^{(\mu +\eta +q)t}I(t)}{e^{(\mu +\eta +q)t}(\mu +\eta +q)}= \frac{q}{\mu +\eta +q}\lim _{t\rightarrow \infty }I(t)=0 \end{aligned} \end{aligned}$$

and

3.14 $$ \begin{aligned} & q(\alpha +\gamma )\lim _{t\rightarrow \infty } \int _{0}^{t}e^{-(\mu +\eta +q)(t-s)} \int _{0}^{s}e^{-\mu (s-u)}I(u)\,du \,ds \\ &\quad = q(\alpha +\gamma )\lim_{t\rightarrow \infty } \int _{0}^{t}I(u)\,du \int _{u}^{t}e^{-(\mu +\eta +q)(t-s)}e^{-\mu (s-u)}\,ds \\ &\quad = \frac{q(\alpha +\gamma )}{\eta +q}\lim_{t \rightarrow \infty }e^{-\mu t} \int _{0}^{t}I(u)e^{\mu u} \bigl(1-e^{-(\eta +q)(t-u)}\bigr)\,du \\ &\quad = \frac{ q(\alpha +\gamma )}{\eta +q}\biggl(\lim_{t \rightarrow \infty }e^{-\mu t} \int _{0}^{t}I(u)e^{-\mu u}\,du-\lim _{t\rightarrow \infty }e^{-t(\mu +\eta +q)} \int _{0}^{t}e^{( \mu +\eta +q)u}I(u)\,du\biggr) \\ &\quad = \frac{ q(\alpha +\gamma )}{\eta +q}\biggl(\lim_{t \rightarrow \infty }\frac{\int _{0}^{t}I(u)e^{\mu u}\,du}{e^{\mu t}} - \lim_{t\rightarrow \infty } \frac{\int _{0}^{t}e^{(\mu +\eta +q)u}I(u)\,du}{e^{(\mu +\eta +q)t}}\biggr) \\ &\quad = \frac{\alpha q}{\mu (\eta +q)}\lim_{t \rightarrow \infty }I(t) - \frac{ q(\alpha +\gamma )}{(\eta +q)(\mu +\eta +q)}\lim _{t \rightarrow \infty }I(t)=0. \end{aligned} $$

From (3.11), (3.12), (3.13), and (3.14), it follows that $\lim_{t\rightarrow \infty }G(t)=0$ a.s.. Therefore, $\lim_{t\rightarrow \infty }S(t)=S^{0} $ a.s..

From (2.12), we easily get $\lim_{t\rightarrow \infty }R(t)= 0$ a.s. Then, by using (2.12), we further obtain

$$ \begin{aligned} \lim_{t\rightarrow \infty }V(t)&= \lim _{t \rightarrow \infty }\biggl(\frac{\Lambda }{\mu }-S(t)-I(t)-R(t)-H_{0}e^{-\mu t}- \alpha \int _{0}^{t}e^{-\mu (t-s)}I(s)\,ds\biggr) \\ &= \frac{\Lambda }{\mu }-S^{0}-\frac{\alpha }{\mu }\lim _{t\rightarrow \infty }I(t)= \frac{\Lambda (\mu \epsilon +q)}{\mu (\mu +\eta +q)}=V^{0} \quad \mbox{a.s.}, \end{aligned} $$

where $H_{0}=\frac{\Lambda }{\mu }-S(0)-I(0)-R(0)-V(0)$. This completes the proof. □

## Permanence in the mean

On the permanence in the mean with probability one for model (1.2) we have the following result.

### Theorem 4.1

*Assume* $\widetilde{R}_{0}>1$. *Then any solution* $(S(t),I(t),R(t),V(t))$ *of model* (1.2) *with initial value* $(S(0),I(0),R(0),V(0)) \in \Gamma $ *is permanent in the mean*. *Namely*, *we have*

$$\begin{aligned} &\liminf_{t\to \infty }\langle S\rangle _{t}\geq \frac{(1-\epsilon )\Lambda }{\mu +q+\beta M_{S}} \quad \textit{a.s.}, \\ &\liminf_{t\to \infty }\langle I)\rangle _{t}\geq \frac{\mu +\alpha +\gamma }{\beta P_{1}\frac{\mu +\alpha +\gamma }{\mu }+\frac{ q(1+\alpha +\gamma ) }{\mu +\eta +q}\sigma ^{2}P_{3}\tilde{\zeta }(S^{0}) +\beta P_{2}+4\sigma ^{2}P_{3}^{2}\tilde{M}}( \tilde{R}_{0}-1)>0 \quad \textit{a.s.}, \\ &\liminf_{t\to \infty }\langle R\rangle _{t}\geq \frac{\gamma }{\mu }T \quad \textit{a.s.}, \\ &\liminf_{t\to \infty }\langle V\rangle _{t}\geq \frac{\epsilon \Lambda }{\mu +\eta } \quad \textit{a.s.}, \end{aligned}$$

*where the constants* $P_{1}$, $P_{2}$, $P_{3}$, *M̃*, $M_{S}$, *and* *T* *will be defined below*.

### Proof

We consider formula (3.1). From (2.9), by the mean value theorem, for any $t\geq 0$, we have

4.1 $$ \begin{aligned} \tilde{\xi }\bigl(S(t),I(t)\bigr)&= \tilde{\zeta }\bigl(S^{0}\bigr)+ \frac{\partial \tilde{\xi }(\xi (t),I(t))}{\partial S}\bigl(S(t)-S^{0} \bigr)+ \frac{\partial \tilde{\xi } (S^{0},\zeta (t)) }{\partial I}I(t) \\ &= \tilde{\zeta }\bigl(S^{0}\bigr)+ \frac{\partial \tilde{\xi }(\xi (t),I(t))}{\partial S} \bigl(H_{1}(t)+G(t)\bigr)+ \frac{\partial \tilde{\xi } (S^{0},\zeta (t)) }{\partial I}I(t) \end{aligned} $$

and

4.2 $$ \begin{aligned} &\bigl(\tilde{\xi }\bigl(S(t),I(t) \bigr)\bigr)^{2}\\ &\quad \leq \bigl( \tilde{\zeta }\bigl(S(t)\bigr) \bigr)^{2} \\ &\quad = \bigl(\tilde{\zeta }\bigl(S^{0}\bigr)\bigr)^{2}+2\tilde{ \zeta }\bigl(S^{0}\bigr) \bigl( \tilde{\zeta }\bigl(S(t)\bigr)-\tilde{ \zeta }\bigl(S^{0}\bigr)\bigr)+\bigl(\tilde{\zeta }\bigl(S(t)\bigr)- \tilde{\zeta }\bigl(S^{0}\bigr)\bigr)^{2} \\ &\quad = \bigl(\tilde{\zeta }\bigl(S^{0}\bigr)\bigr)^{2}+2\tilde{ \zeta }\bigl(S^{0}\bigr) \frac{\partial \tilde{\zeta } (\xi (t))}{\partial S}\bigl(S(t)-S^{0} \bigr)+\biggl( \frac{\partial \tilde{\zeta } (\xi (t))}{\partial S}\biggr)^{2}\bigl(S(t)-S^{0} \bigr)^{2} \\ &\quad = \bigl(\tilde{\zeta }\bigl(S^{0}\bigr)\bigr)^{2}+2\tilde{ \zeta }\bigl(S^{0}\bigr) \frac{\partial \tilde{\zeta } (\xi (t))}{\partial S}\bigl(H_{1}(t)+G(t) \bigr)+\biggl( \frac{\partial \tilde{\zeta } (\xi (t))}{\partial S}\biggr)^{2}\bigl(H_{1}(t)+G(t) \bigr)^{2}, \end{aligned} $$

where $\xi (t)$ is situated between $S^{0}$ and $S(t)$ and $\zeta (t)\in (0,I(t))$. According to Lemma 3 given in [34], we obtain

$$ P_{1}=\max_{\Gamma }\biggl\{ \biggl\vert \frac{\partial \tilde{\xi }(S,I)}{\partial S} \biggr\vert \biggr\} < \infty ,\qquad P_{2}=\max _{\Gamma }\biggl\{ \biggl\vert \frac{\partial \tilde{\xi } (S^{0},I) }{\partial I} \biggr\vert \biggr\} < \infty $$

and

$$ P_{3}=\max_{\Gamma }\biggl\{ \biggl\vert \frac{\partial \tilde{\zeta } (S)}{\partial S} \biggr\vert \biggr\} < \infty . $$

From (3.1) and combining with (4.1) and (4.2), we can obtain

4.3 $$ \begin{aligned} \frac{1}{t} \log I(t)&\geq \frac{\log I(0)}{t}+\frac{1}{t} \int _{0}^{t}\biggl[ \beta \tilde{\zeta } \bigl(S^{0}\bigr)-( \mu +\gamma +\alpha )-\frac{\sigma ^{2}}{2}\bigl( \tilde{\zeta }\bigl(S^{0}\bigr)\bigr)^{2}\biggr]\,ds \\ &\quad{}+\frac{\beta }{t} \int _{0}^{t} \frac{\partial \tilde{\xi }(\xi (s),I(s))}{\partial S}\bigl(H_{1}(s)+G(s) \bigr)\,ds+ \frac{\beta }{t} \int _{0}^{t} \frac{\partial \tilde{\xi } (S^{0},\zeta (s)) }{\partial I}I(s)]\,ds \\ &\quad{}-\frac{\sigma ^{2}}{t} \int _{0}^{t} \tilde{\zeta }\bigl(S^{0} \bigr) \frac{\partial \tilde{\zeta } (\xi (s))}{\partial S}\bigl(H_{1}(s)+G(s)\bigr)\,ds \\ &\quad{}-\frac{\sigma ^{2}}{t} \int _{0}^{t} \biggl( \frac{\partial \tilde{\zeta } (\xi (s))}{\partial S} \biggr)^{2}\bigl(H_{1}^{2}(s)+G^{2}(s) \bigr)\,ds + \frac{\sigma }{t} \int _{0}^{t}\tilde{\xi }(S(s),I(s)\,dB(s). \end{aligned} $$

We then compute to obtain

4.4 $$\begin{aligned}& \begin{aligned} \langle G\rangle _{t} &\geq -\frac{1}{t} \int _{0}^{t}I(u)\,du- \frac{(\alpha +\gamma )}{t} \int _{0}^{t} \int _{0}^{u}e^{-\mu (u-s)}I(s)\,ds \,du \\ &\geq -\frac{1}{t} \int _{0}^{t}I(u)\,du- \frac{(\alpha +\gamma )}{\mu } \frac{1}{t} \int _{0}^{t}I(u)\,du=- \frac{\mu +\alpha +\gamma }{\mu }\langle I \rangle _{t}, \end{aligned} \end{aligned}$$

4.5 $$\begin{aligned}& \begin{aligned} \langle G\rangle _{t}&\leq \frac{ q}{t} \int _{0}^{t} \int _{0}^{u}e^{-(\mu +\eta +q)(u-s)}I(s)\,ds \,du \\ &\quad {}+ \frac{ q(\alpha +\gamma )}{t} \int _{0}^{t} \int _{0}^{u}e^{-( \mu +\eta +q)(u-s)} \int _{0}^{s}e^{-\mu (s-w)}I(w)\,dw \,ds \,du \\ &\leq \frac{q(1+\alpha +\gamma )}{\mu +\eta +q}\langle I \rangle _{t}, \end{aligned} \end{aligned}$$

and

4.6 $$ \begin{aligned} \bigl\langle G^{2}\bigr\rangle _{t}&\leq \frac{4}{t} \int _{0}^{t}\biggl[I^{2}(u)+( \alpha + \gamma )^{2}\biggl( \int _{0}^{u}e^{-\mu (u-s)}I(s)\,ds \biggr)^{2}+q^{2}\biggl( \int _{0}^{u}e^{-(\mu +\eta +q)(u-s)}I(s)\,ds \biggr)^{2} \\ &\quad{}+\bigl[q(\alpha +\gamma )\bigr]^{2}\biggl( \int _{0}^{u}e^{-(\mu +\eta +q)(u-s)} \int _{0}^{s}e^{-\mu (s-w)}I(w)\,dw \,ds \biggr)^{2}\biggr]\,du \\ &\leq \frac{4}{t} \int _{0}^{t}\biggl[\frac{\Lambda }{\mu }I(u)+ \frac{(\alpha +\gamma )^{2}\Lambda }{\mu ^{2}} \int _{0}^{u}e^{-\mu (u-s)}I(s)\,ds \\ &\quad{}+\frac{q^{2}\Lambda }{\mu (\mu +\eta +q)} \int _{0}^{u}e^{-(\mu +\eta +q)(u-s)}I(s)\,ds \\ &\quad{}+ \frac{q^{2}(\alpha +\gamma )^{2}\Lambda }{\mu ^{2}(\mu +\eta +q)} \int _{0}^{u} e^{-(\mu _{+}\eta +q)(u-s)} \int _{0}^{s}e^{-\mu (s-w)}I(w)\,dw \,ds\biggr] \,du \\ &\leq 4\tilde{M}\langle I\rangle _{t}, \end{aligned} $$

where

$$ \tilde{M}=\frac{\Lambda }{\mu }+ \frac{(\alpha +\gamma )^{2}\Lambda }{\mu ^{3}} + \frac{q^{2}\Lambda }{\mu (\mu +\eta +q)^{2}}+ \frac{q^{2}(\alpha +\gamma )^{2}\Lambda }{\mu ^{2}(\mu +\eta +q)^{2}}. $$

From (4.4), (4.5), and (4.6), we can rewrite (4.3) as

4.7 $$ \begin{aligned} \frac{ \log I(t)}{t} &\geq \frac{\log I(0)}{t}+\biggl[\beta \tilde{\zeta }\bigl(S^{0}\bigr)-(\mu + \gamma +\alpha )- \frac{\sigma ^{2}}{2}\bigl(\tilde{\zeta }\bigl(S^{0} \bigr)\bigr)^{2}\biggr] \\ &\quad{}-\beta P_{1}\frac{\mu +\alpha +\gamma }{\mu }\langle I \rangle _{t}- \frac{ q(\mu +\alpha +\gamma ) \sigma ^{2} P_{3}}{\mu (\mu +\eta +q)} \tilde{\zeta }\bigl(S^{0}\bigr)\langle I\rangle _{t}- \beta P_{2}\langle I \rangle _{t} \\ &\quad{}-4\sigma ^{2}P_{3}^{2}\tilde{M}\langle I\rangle _{t}+ \Psi (t), \end{aligned} $$

where

$$ \Psi (t)=\frac{\ln I(0)}{t} -\beta P_{1}\langle H_{1} \rangle _{t}- \sigma ^{2} P_{3}\tilde{\zeta } \bigl(S^{0}\bigr)\langle H_{1}\rangle _{t}- \sigma ^{2}P_{3}^{2}\bigl\langle H_{1}^{2} \bigr\rangle _{t}+\frac{\sigma }{t} \int _{0}^{t}\tilde{\xi }\bigl(S(s),I(s)\bigr) \,dB(s). $$

According to L’Hospital’s rule, we get

4.8 $$ \begin{aligned} \lim_{t\rightarrow \infty }\langle H_{1}\rangle _{t}&= \lim_{t\rightarrow \infty }\biggl\{ \frac{1}{t} \int _{0}^{t}\biggl[ \tilde{\eta }e^{-(\mu +\eta +q)s} + \tilde{H}_{0}e^{-\mu s} \frac{\eta }{q +\eta }-\biggl(V(0)- \frac{\epsilon \Lambda }{\mu +\eta }\biggr)e^{-(\mu +\eta +q)s} \\ &\quad{}- \tilde{H}_{0}e^{-(\mu +\eta +q)s}\frac{q}{\eta +q}\biggr]\,ds \biggr\} \\ &= \tilde{\eta }\lim_{t\rightarrow \infty } \frac{1-e^{-(\mu +\eta +q)t}}{t(\mu +\eta +q)}+ \tilde{H}_{0} \frac{\eta }{q+\eta }\lim_{t\rightarrow \infty } \frac{1-e^{-\mu t}}{\mu t} \\ &\quad{}-\frac{1}{\mu +\eta +q}\biggl(V(0)- \frac{\epsilon \Lambda }{\mu +\eta }\biggr)\lim _{t\rightarrow \infty } \frac{1-e^{-(\mu +\eta +q)t}}{t} \\ &\quad{}- \frac{q}{(\eta +q)(\eta + q+\mu )}\tilde{H}_{0}\lim_{t\rightarrow \infty } \frac{1-e^{-(\mu +\eta +q)t}}{t}\\ &= 0 \end{aligned} $$

and

4.9 $$ \begin{aligned} \lim_{t\rightarrow \infty }\bigl\langle H_{1}^{2}\bigr\rangle _{t}&\leq \lim _{t\rightarrow \infty }\biggl\{ \frac{4}{t} \int _{0}^{t}\biggl[ \tilde{\eta }^{2} e^{-2(\mu +\eta +q)s} +\tilde{H}_{0}^{2}e^{-2\mu s}\biggl( \frac{\eta }{q +\eta }\biggr)^{2} \\ &\quad{}+ \biggl(V(0)-\frac{\epsilon \Lambda }{\mu +\eta }\biggr)^{2}e^{-2( \mu +\eta +q)s} + \tilde{H}_{0}^{2}e^{-(\mu +\eta +q)s}\biggl( \frac{q}{\eta +q} \biggr)^{2}\biggr]\,ds \biggr\} \\ &= 0. \end{aligned} $$

From equations (4.8) and (4.9), by the strong law of large numbers, we obtain that $\lim_{t\to \infty }\Psi (t)=0 $ a.s.. Therefore, we finally have

4.10 $$ \liminf_{t\to \infty }\langle I\rangle _{t} \geq \frac{\beta \tilde{\zeta }(S^{0})-(\mu +\gamma +\alpha )-\frac{\sigma ^{2}}{2}(\tilde{\zeta }(S^{0}))^{2}}{\beta P_{1}\frac{\mu +\alpha +\gamma }{\mu }+\frac{ q(1+\alpha +\gamma ) }{\mu +\eta +q}\sigma ^{2}P_{3}\tilde{\zeta }(S^{0})+\beta P_{2}+4\sigma ^{2}P_{3}^{2}\tilde{M}}:=T>0 \quad \mbox{a.s..} $$

Next, we prove the permanence in the mean of $S(t)$. The proof is similar to [34]. According to Lemma 3 given in [34], we have $M_{S}\triangleq \max_{\Gamma }\{\frac{f(S,I)}{S}\}<\infty $. Integrating the first equation of model (1.2) from 0 to *t*, we can obtain

$$ \frac{S(t)-S(0)}{t}\geq (1-\epsilon )\Lambda -\frac{1}{t} \int _{0}^{t}[ \beta M_{S}+\mu +q]S(s) \,ds-\frac{\sigma }{t} \int _{0}^{t}f\bigl(S(s),I(s)\bigr)\,dB(s). $$

Taking $t\to \infty $ and the strong law of large numbers, we further have

$$ \liminf_{t\to \infty }\langle S\rangle _{t}\geq \frac{(1-\epsilon )\Lambda }{\mu +q+\beta M_{S}} \quad \mbox{a.s..} $$

Secondly, from (4.10) and integrating the third equation of model (1.2) from 0 to *t*, we can obtain

$$ \liminf_{t\to \infty }\langle R\rangle _{t}\geq \frac{\gamma }{\mu }T \quad \mbox{a.s..} $$

Lastly, integrating the last equation of model (1.2), we obtain

$$ \frac{V(t)-V(0)}{t}\geq \epsilon \Lambda -\frac{1}{t} \int _{0}^{t}[ \mu +\eta ]V(s)\,ds. $$

Therefore, taking $t\to \infty $, we finally have

$$ \liminf_{t\to \infty }\langle V\rangle _{t}\geq \frac{\epsilon \Lambda }{\mu +\eta } \quad \mbox{a.s..} $$

This completes the proof. □

### Remark 4.2

Comparing $R_{0}=\frac{\beta \tilde{\zeta }(S^{0})}{\mu +\gamma +\alpha }$ with $\tilde{R}_{0}$, we can easily see that $\tilde{R}_{0}< R_{0}$ for any $\sigma >0$ and if $\sigma =0$, then $\tilde{R}_{0}= R_{0}$. Namely, the environmental noise can greatly change the properties of an epidemic model.

## Numerical simulation

In this section we analyze the stochastic behavior of model (1.2) by means of the numerical simulations in order to make readers understand our results better. The numerical simulation method can be found in [32]. The corresponding discretization system of model (1.2) is given as follows:

$$ \textstyle\begin{cases} S_{k+1}= S_{k}+[(1-q)\Lambda - \beta f(S_{k},I_{k})-( \mu +p) S_{k}+\delta V_{k}]\Delta t \\\hphantom{S_{k+1}=}{} +f(S_{k},I_{k})[\sigma \xi _{k}\sqrt{\Delta t}+ \frac{1}{2}\sigma ^{2}(\xi _{k}^{2}-1)\Delta t], \\ I_{k+1}= I_{k}+[\beta f(S_{k},I_{k})-( \mu +\gamma +\alpha )I_{k}]\Delta t \\\hphantom{I_{k+1}=}{} +f(S_{k},I_{k})[\sigma \xi _{k}\sqrt{\Delta t}+ \frac{1}{2}\sigma ^{2}(\xi _{k}^{2}-1)\Delta t], \\ R_{k+1}= R_{k}+[\gamma I_{k}- \mu R_{k}] \Delta t, \\ V_{k+1}= V_{k}+[q\Lambda +pS_{k} - (\mu + \alpha ) V_{k}]\Delta t, \end{cases} $$

where $\xi _{k}$ ($k=1,2,\ldots $) are the Gaussian random variables which follow the standard normal distribution $N(0,1)$.

### Example 1

In model (1.2), we take $f(S,I)=\frac{SI}{N}$ (standard incidence).

**Figure 1:**
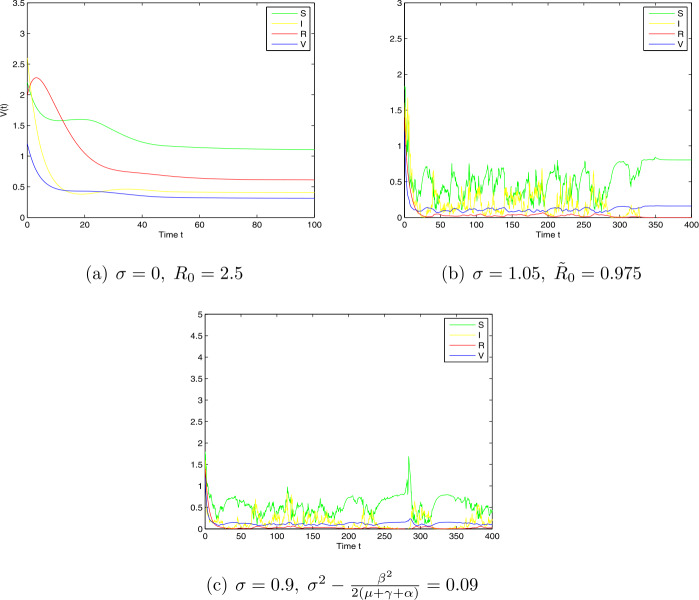
Simulation for paths $S(t)$, $I(t)$, $R(t)$, and $V(t)$ for the stochastic system and the corresponding deterministic system with initial value $(S(0),I(0),R(0),V(0))=(0.5,0.06,0.5,0.5)$ and step size $\Delta t=0.001$

*Case 1.* (i) Choose $\Lambda =0.2$, $\beta =0.99$, $\mu =0.1$, $\gamma =0.15$, $\sigma =1.05$, $p= 0.07$, $\delta =0.6$, $q=0.08$, and $\alpha =0.2$. For deterministic model (1.1), $R_{0}=2.5>1$. From the numerical simulations (see Fig. 1.a), it is clear that the endemic equilibrium $E^{*}$ is also globally asymptotically stable if only the basic reproduction number $R_{0}$ is greater than one. For the corresponding stochastic model (1.2), we have $\tilde{R}_{0}= 0.975<1$, which is the case of Theorem 3.1. From the numerical simulations, we see that the disease will die out (see Fig. 1.b).

(ii) Choose $\Lambda =3.4$, $\beta =1.2$, $\mu =0.2$, $\gamma =0.4$, $\sigma =0.9$, $p= 0.07$, $\delta =0.6$, $q=0.08$, and $\alpha =0.4$. By computing, we have $\sigma ^{2}-\frac{\beta ^{2}}{2(\mu +\gamma +\alpha )}=0.09> 0$, which is the case of Theorem 3.1. From the numerical simulations, we see that the disease will die out (see Fig. 1.c).

**Figure 2:**
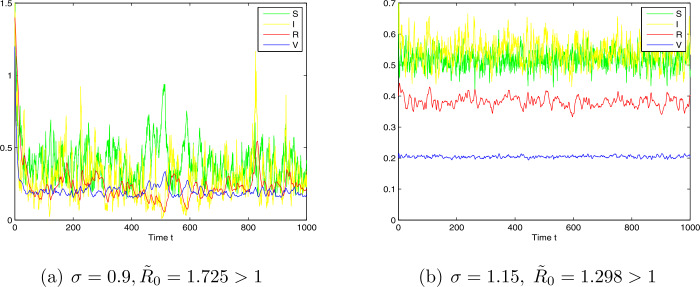
Simulation for paths $S(t)$, $I(t)$, $R(t)$, and $V(t)$ for the stochastic system with initial value $(S(0),I(0),R(0),V(0))=(0.5,0.06,0.5,0.5)$ for different noise intensities $\sigma =1.15$ (**a**) and $\sigma =0.9$ (**b**)

*Case 2.* Choose $\Lambda =3.4$, $\beta =1.2$, $\mu =0.1$, $\gamma =0.3$, $\sigma =1.15$, $p= 0.02$, $\delta =0.6$, $q=0.01$, and $\alpha =0.2$. By computing, we have $R_{0}=2.4$, $\tilde{R}_{0}=1.298>1$. The numerical simulations are given in Figs. 2.a and 2.b, which show that model (1.2) is permanent in the mean with probability one.

### Example 2

In model (1.2), take $f(S,I)=h(S)g(I)=\frac{SI}{1+\omega I^{2}}$, where *ω* is a positive constant.

**Figure 3:**
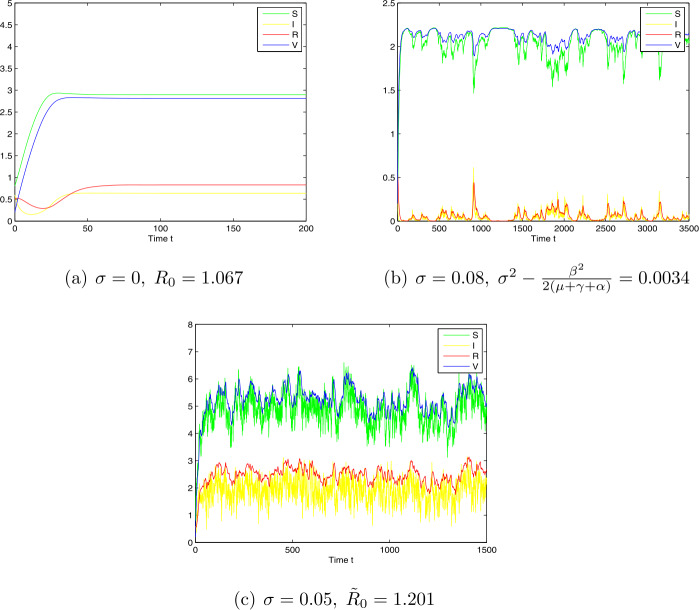
Simulation for paths $S(t)$, $I(t)$, $R(t)$, and $V(t)$ for the stochastic system and the corresponding deterministic system with initial value $(S(0),I(0),R(0),V(0))=(0.5,0.06,0.5,0.5)$ and step size $\Delta t=0.001$

*Case 1.* Choose $\Lambda =0.4$, $\beta =0.04$, $\mu =0.1$, $\gamma =0.03$, $\sigma =0.08$, $\alpha =0.02$, $p=0.02$, $q=0.01$, $\omega =1$, and $\delta =0.16$. By computing, we have $\sigma ^{2}-\frac{\beta ^{2}}{2(\mu +\gamma +\alpha )}= 0.0034 >0$, which is the case of Theorem 3.1. From the numerical simulations given in Fig. 3.b, we see that disease will die out.

*Case 2.* Choose $\Lambda =0.4$, $\beta =0.2$, $\mu =0.1$, $\gamma =0.3$, $\sigma =0.05$, $\alpha =0.2$, $p=0.02$, $q=0.01$, $\omega =1$, and $\delta =0.16$. We have $R_{0}= 1.23$, $\tilde{R}_{0}= 1.201>1 $. The numerical simulations found in Fig. 3.c show that model (1.2) is permanent in the mean with probability one.

### Example 3

In model (1.2), take $f(S,I)=\frac{SI}{1+\omega _{1}S+\omega _{2}I}$ (Beddington–DeAngelis incidence), where $\omega _{1}$ and $\omega _{2}$ are nonnegative constants.

**Figure 4:**
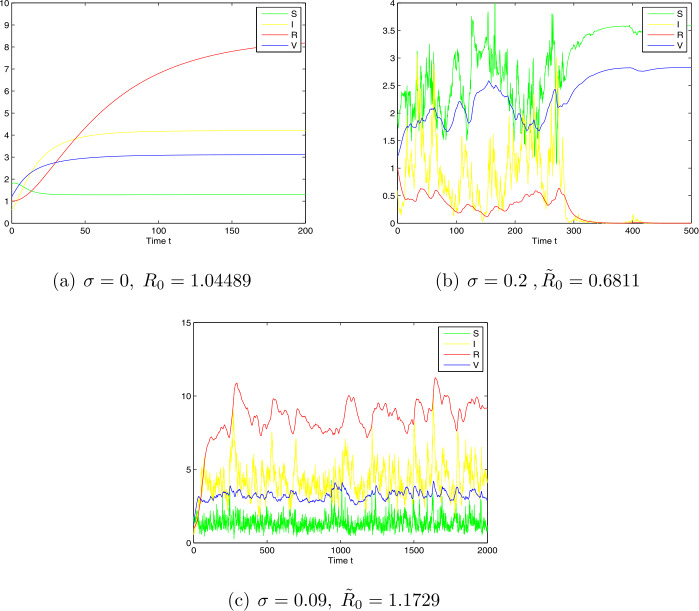
Simulation for paths $S(t)$, $I(t)$, $R(t)$, and $V(t)$ for the stochastic system and the corresponding deterministic system with initial value $(S(0),I(0),R(0),V(0))=(0.5,0.06,0.5,0.5)$ and step size $\Delta t=0.001$

*Case 1.* Choose $\Lambda =0.4$, $\beta =0.1$, $\mu =0.08$, $\gamma =0.03$, $\sigma =0.2$, $\alpha =0.02$, $\delta =0.06$, $q=0.08$, $p= 0.07$, $m=0.1$, and $n=0.5$. By computing we have $R_{0}=1.04489$, $\tilde{R}_{0}= 0.6811 <1$, which is the case of Theorem 3.1. From the numerical simulations given in Fig. 4.b, we see that the disease will die out.

*Case 2.* Choose $\Lambda =0.4$, $\beta =0.2$, $\mu =0.02$, $\gamma =0.04$, $\sigma =0.09$, $\alpha =0.02$, $\delta =0.06$, $q=0.08$, $p= 0.07$, $m=0.1$, and $n=0.5$. By computing, we have $R_{0}=1.176$, $\tilde{R}_{0}=1.1729 >1$. The numerical simulations given in Fig. 4.c show that model (1.2) is permanent in the mean with probability one.

## Conclusion

Environmental noises have a critical influence on the development of an epidemic. In this paper, we study the dynamics of a stochastic SIRV model with general nonlinear incidence rate. We assume that the stochastic perturbation is a white noise type which perturbs the disease transmission coefficient *β*. This is a well-established way of introducing stochastic environmental noise into biologically realistic population dynamic models that were used in [44, 46].

The value of our study lies in two aspects: Mathematically, we show that the global dynamics of deterministic model (1.1) can be governed by its reproduction number $R_{0}$, while the dynamics of its stochastic version (1.2) is seen to be governed by $\widetilde{R}_{0}$. In addition, we have provided the analytic results on the existence of the global positive solution, the extinction (i.e.,disease-free) or persistence (i.e.,endemic) of the disease for stochastic model (1.2).

Epidemiologically, we summarize our main findings as follows:

1. *Noise can suppress the disease outbreak*: Theorem 3.1 (a) indicates that the extinction of the disease in stochastic model (1.2) occurs if the basic reproduction number $\widetilde{R}_{0}= \frac{\beta \tilde{\zeta }(S^{0})}{\mu +\gamma +\alpha } - \frac{\sigma ^{2}(\tilde{\zeta }(S^{0}))^{2}}{2(\mu +\gamma +\alpha )}<1$. We show that deterministic model (1.1) admits a unique endemic equilibrium $E^{*}$ which is globally asymptotically stable if its basic reproduction number $R_{0}>1$. Notice that $\widetilde{R}_{0}< R_{0}$, and it is possible that $\widetilde{R}_{0}<1<R_{0}$. This is the case when deterministic model (1.1) has an endemic (see Fig. 1a) while stochastic model (1.2) has disease extinction with probability one (see Fig. 1b). This implies that large noise intensities can inhibit the spread of a disease, which means the random perturbations can change the disease dynamics.

2. *The effects of the intensity of noise level*: From part (b) of Theorem 3.2, under large noise intensity case, i.e., the condition $\sigma ^{2}>\frac{\beta ^{2}}{2(\mu +\gamma +\alpha )}$ holds, the disease will become extinct exponentially. In other words, in the case of sufficiently large noise, we should use the stochastic model rather than the deterministic model to describe the population dynamics (see Fig. 1.c). One can know that if $R_{0}>1$, model(1.1) admits a globally stable endemic equilibrium $E^{*}$. In this case, when the noise intensity *σ* is small enough to imply that $\widetilde{R}_{0}>1$ from Theorem 4.1, one can know that the stochastic model preserves the property of the global stability, and the noise can force the solutions of model (1.2) to oscillate strongly around the endemic point (see Figs. 2.a and 2.b). In addition, from Figs. 2.a and 2.b, one can observe the effects of increasing noise intensity *σ* on the increased level of non-equilibrium fluctuation in the stochastic dynamics of model (1.2).

Furthermore, from Theorems 3.1, 4.1 and numerical simulation results (e.g., Figs. 1–4), we can conclude that, when the intensity of noise is small, the stochastic model preserves the property of the global stability. In this case, we can ignore noise and use the deterministic model to approximate the population dynamics. However, the large intensity of noise can force the solution of model (1.2) to oscillate strongly around the disease-free or endemic points, or the extinction. In these cases, we cannot ignore the effect of noise and, therefore, we cannot use the deterministic model but the stochastic model to describe the disease dynamics.

Some interesting topics deserve further investigations. SDEs are being increasingly used in a wide range of areas, for example, finance and biology. There has recently been a large explosion in the number of papers using SDEs to model how diseases spread. However, these papers introduce stochasticity in a different way by parameter perturbation, which is appropriate if one of the parameters is a random variable. Another way to introduce stochasticity into deterministic models is telegraph noise where the parameters switch from one set to another according to a Markov switching process. Therefore, we may study a stochastic version of model (1.2) including Markovian switching into all parameters. These studies are in progress.

## Data Availability

Data sharing not applicable to this article as no datasets were generated or analysed during the current study.
